# Nitric Oxide-Dependent Mechanisms Underlying MK-801- or Scopolamine-Induced Memory Dysfunction in Animals: Mechanistic Studies

**DOI:** 10.3390/ijms222212282

**Published:** 2021-11-13

**Authors:** Paulina Cieślik, Anna Siekierzycka, Adrianna Radulska, Agata Płoska, Grzegorz Burnat, Piotr Brański, Leszek Kalinowski, Joanna M. Wierońska

**Affiliations:** 1 Department of Neurobiology, Maj Institute of Pharmacology, Polish Academy of Sciences, 12 Smętna Street, 31-343 Kraków, Poland; cieslik@if-pan.krakow.pl (P.C.); anna.siekierzycka@gumed.edu.pl (A.S.); burnat@if-pan.krakow.pl (G.B.); nfbransk@cyf-kr.edu.pl (P.B.); 2 Department of Medical Laboratory Diagnostics—Fahrenheit Biobank BBMRI.pl, Medical University of Gdańsk, 7 Dębinki Street, 80-211 Gdańsk, Poland; adrianna.radulska@gumed.edu.pl (A.R.); agata.ploska@gumed.edu.pl (A.P.); 3 Biobanking and Biomoleclular Resources Research Infrastructure Consortium Poland (BBMRI.pl), 7 Dębinki Street, 80-211 Gdańsk, Poland; 4 BioTechMed Centre, Department of Mechanics of Materials and Structures, University of Technology, 11/12 Narutowicza, 80-233 Gdańsk, Poland

**Keywords:** MK-801, scopolamine, L-arginine, cGMP, S-nitrosylation, schizophrenia, Alzheimer’s disease, DDAH1, ADMA, SDAMA, NMMA

## Abstract

MK-801, an NMDA receptor antagonist, and scopolamine, a cholinergic receptor blocker, are widely used as tool compounds to induce learning and memory deficits in animal models to study schizophrenia or Alzheimer-type dementia (AD), respectively. Memory impairments are observed after either acute or chronic administration of either compound. The present experiments were performed to study the nitric oxide (NO)-related mechanisms underlying memory dysfunction induced by acute or chronic (14 days) administration of MK-801 (0.3 mg/kg, i.p.) or scopolamine (1 mg/kg, i.p.). The levels of L-arginine and its derivatives, L-citrulline, L-glutamate, L-glutamine and L-ornithine, were measured. The expression of constitutive nitric oxide synthases (cNOS), dimethylaminohydrolase (DDAH1) and protein arginine N-methyltransferases (PMRTs) 1 and 5 was evaluated, and the impact of the studied tool compounds on cGMP production and NMDA receptors was measured. The studies were performed in both the cortex and hippocampus of mice. S-nitrosylation of selected proteins, such as GLT-1, APP and tau, was also investigated. Our results indicate that the availability of L-arginine decreased after chronic administration of MK-801 or scopolamine, as both the amino acid itself as well as its level in proportion to its derivatives (SDMA and NMMA) were decreased. Additionally, among all three methylamines, SDMA was the most abundant in the brain (~70%). Administration of either compound impaired eNOS-derived NO production, increasing the monomer levels, and had no significant impact on nNOS. Both compounds elevated DDAH1 expression, and slight decreases in PMRT1 and PMRT5 in the cortex after scopolamine (acute) and MK-801 (chronic) administration were observed in the PFC, respectively. Administration of MK-801 induced a decrease in the cGMP level in the hippocampus, accompanied by decreased NMDA expression, while increased cGMP production and decreased NMDA receptor expression were observed after scopolamine administration. Chronic MK-801 and scopolamine administration affected S-nitrosylation of GLT-1 transport protein. Our results indicate that the analyzed tool compounds used in pharmacological models of schizophrenia or AD induce changes in NO-related pathways in the brain structures involved in cognition. To some extent, the changes resemble those observed in human samples.

## 1. Introduction

Learning and memory processes are crucial for the interaction of individuals with their environments and involve the coordination of large, widely distributed brain networks.

Memory is often understood as an information processing system with explicit and implicit functioning that comprises a sensory processor, short-term memory (STM) and long-term memory (LTM) [1]. The sensory processor retains information derived from the senses—it is an automatic response and beyond cognitive control [2]. Short-term memory allows the storage of a small amount of information for a period of several seconds [1]. In contrast, long-term memory, once consolidated, can store much larger quantities of information and is persistent and stable for a potentially unlimited duration [1].

Different brain areas are involved in the neuroanatomy of memory, and each of them is presumed to be involved in specific types of memory. The hippocampus is believed to be involved in spatial and declarative learning [3], while the prefrontal cortex (PFC, a part of the frontal lobe) maintains representations of task-relevant stimuli, decision making, short-term memory and working memory [4,5,6]. In contrast to STM, LTM is maintained by stable and permanent changes in neural connections that are widely distributed throughout the brain. Consolidation of STM into LTM at the molecular level presumably involves synaptic consolidation and system consolidation. The former involves alterations in synaptic protein synthesis, activation of the intracellular transduction cascade and changes in membrane potential [7]. Synaptic plasticity and synaptic strength, which underlie memory formation, are regulated by long-term potentiation (LTP), which is considered to be an important mechanism in learning and memory processes [7]. According to the theory of systems consolidation, when novel information is originally encoded and registered, the memory of these new stimuli is retained in both the hippocampus and cortical regions [8]. The hippocampus is essential to the consolidation of information from STM to LTM [8].

Learning and memory processes are widely studied in animals, primarily because of the need to search for new drug targets, but also to understand the mechanisms underlying memory impairments. Commonly used compounds to induce memory impairments are NMDA (N-methyl-D-aspartic acid) receptor antagonists (e.g., MK-801) and scopolamine, a non-selective cholinergic receptor antagonist. The compounds induce schizophrenia-related [9,10] or Alzheimer-related [11,12,13] cognitive decline respectively, by influencing a variety of cellular and synaptic mechanisms. Nitric oxide (NO)-related pathways are among the factors that are essential in memory processes due to their regulation of NMDA-dependent cGMP (cyclic guanosine monophosphate) synthesis, protein activity (through the process of S-nitrosylation) and LTP [14,15,16].

In the present study, a complex analysis of the impact of acute and chronic (14 days) administration of both MK-801 and scopolamine on NO-related pathways was performed. The following parameters were measured: the levels of L-arginine and its derivatives, nitric oxide synthases (nNOS and eNOS), dimethylarginine dimethylaminohydrolase 1 (DDAH1), protein arginine N-methyltransferases 1 and 5 (PMRT1 and PMRT5), NMDA and cGMP levels and S-nitrosylation of selected proteins. The analysis was performed in the prefrontal cortices (PFC) and hippocampi of MK-801- and scopolamine-treated animals.

## 2. Results

### 2.1. Experimental Design

The tool compounds MK-801 and scopolamine were administered in two schedules:

- Acute administration at doses of 0.3 and 1 mg/kg respectively, and the PFCs or hippocampi were dissected 30 min after the administration.
- Chronic administration for 14 days at doses of 0.3 mg/kg (MK-801) and 1 mg/kg (scopolamine), and the PFCs or hippocampi were dissected 24 h after the last administration.

The doses of the compounds were adjusted according to previous studies. MK-801 at the dose of 0.3 mg/kg induced cognitive deficits in CD1 mice both after acute and prolonged administration [17,18,19,20,21], and scopolamine was used at the dose of 1 mg/kg [22].

The following parameters were measured:

1. The levels of selected amino acids: L-citrulline, L-glutamate, L-glutamine, L-ornithine and L-arginine and its derivatives: asymmetric dimethylarginine (ADMA), symmetric dimethylarginine (SDMA) and n-monomethyl-L-arginine (NMMA), measured by mass spectrometry.
2. L-Arg/ADMA, L-Arg/SDMA and L-Arg/NMMA ratios.
3. The expression of eNOS, nNOS, DDAH1, PMRT1 and PMRT5, analyzed by Western blotting.
4. GluN2B subunit-containing NMDA receptor and cGMP levels, analyzed by Western blotting and ELISA, respectively.
5. S-nitrosylation of selected proteins: Tau, apolipoprotein (APP) and GLT-1 transporter (glutamate transporter 1).

### 2.2. Acute MK-801 or Scopolamine Administration

#### 2.2.1. Amino Acids in PFC and Hippocampus

The concentrations of amino acids in the control group PFC were as follows: 18.6 µM L-arginine, 3.65 µM L-citrulline, 1303 µM L-glutamate, 743.5 µM L-glutamine and 3.8 µM L-ornithine. The same trend but with slightly lower concentrations was noted in the hippocampus: 16.5 µM L-arginine, 3.54 µM L-citrulline, 990 µM L-glutamate, 547.74 µM L-glutamine and 3.8 µM L-ornithine.

**Figure 1:**
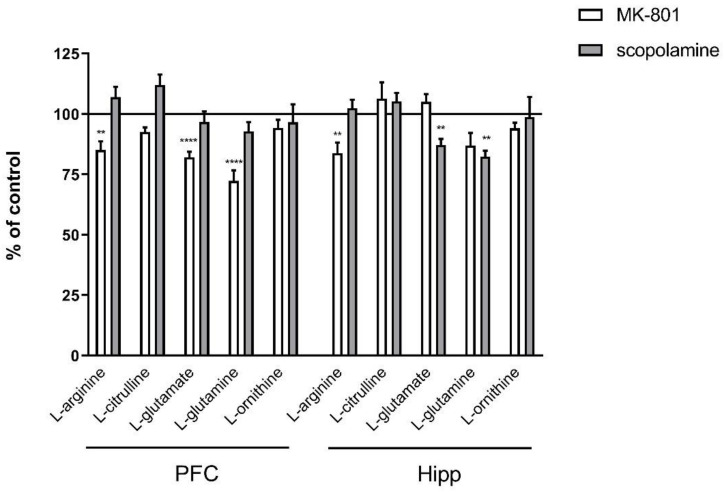
Amino acid levels in the prefrontal cortex (PFC) and hippocampus (Hipp) after acute administration of MK-801 (0.3 mg/kg) or scopolamine (1 mg/kg). Data are presented as means ± SEM. ** *p* < 0.01; **** *p* < 0.0001 vs. control.

In the PFC, acute administration of MK-801 induced significant decreases in L-arginine (F_(2.27)_ = 5.96; *p* < 0.007), L-glutamate (F_(2.25)_ = 17.51, *p* < 0.0001) and L-glutamine (F_(2.27)_ = 18.8, *p* < 0.0001). Acute scopolamine administration induced no changes in the levels of investigated amino acids (Figure 1). 

In the hippocampus, acute administration of MK-801 induced a significant decrease in L-arginine (F_(2.26)_ = 8.16; *p* < 0.001). No changes in L-citrulline or L-ornithine levels were observed. Acute administration of scopolamine induced a significant decrease in L-glutamate and L-glutamine (F_(2.27)_ = 15.59, *p* < 0.0001 and F_(2.27)_ = 8.72, *p* < 0.001, respectively) (Figure 1).

#### 2.2.2. L-Arginine Derivatives in PFC and Hippocampus

In the PFC of control animals, SDMA constituted ca. 60% (0.21 µg/g of tissue) of L-arginine derivatives, followed by ADMA at 26% (0.09 µg/g of tissue) and NMMA at 14% (0.05 µg/g of tissue). The same tendency was observed in the hippocampus, where SDMA accounted for 56% (0.19 µg/g of tissue), ADMA accounted for 27%(0.09 µg/g of tissue) and NMMA accounted for 17% (0.059 µg/g of tissue).

**Figure 2:**
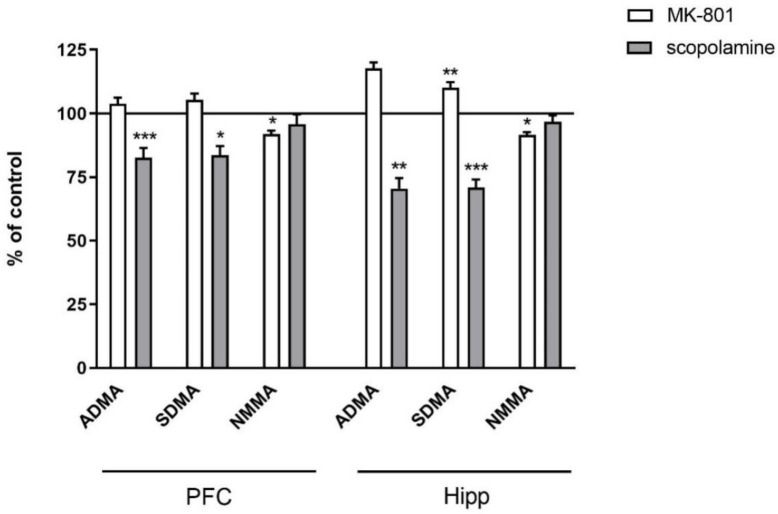
ADMA, SDMA and NMMA levels in the prefrontal cortex (PFC) and hippocampus (Hipp) after acute administration of MK-801 (0.3 mg/kg) or scopolamine (1 mg/kg). Data are presented as means ± SEM. * *p* < 0.05; ** *p* < 0.01; *** *p* < 0.001 vs. control.

In the PFC, acute administration of MK-801 did not induce any significant changes in ADMA or SDMA, and decreased the level of NMMA (F_(2.27)_ = 9.9, *p* < 0.0006). Acute administration of scopolamine decreased the level of ADMA (F_(2.26)_ = 3.74, *p* < 0.03) and SDMA (F_(2.27)_ = 3.39, *p* < 0.04) (Figure 2).

In the hippocampus, acute MK-801 injection induced an increase in the SDMA level (F_(2.27)_ = 15.38, *p* < 0.0001) and a decrease in NMMA (F_(2.27)_ = 6.6, *p* < 0.007). Acute scopolamine administration induced decreases in ADMA (F_(2.25)_ =8.14, *p* < 0.001) and SDMA (F_(2.25)_ = 15.34, *p* < 0.0001). No changes in the NMMA level were detected (Figure 2).

**Figure 3:**
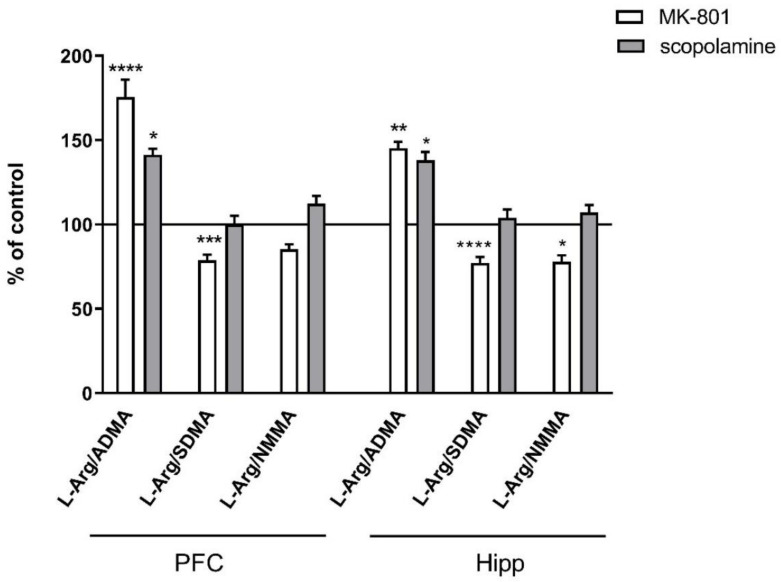
L-arginine (L-Arg)/ADMA, L-arginine/SDMA and L-arginine/NMMA ratios in the prefrontal cortex (PFC) and hippocampus (Hipp) after acute administration of MK-801 (0.3 mg/kg) or scopolamine (1 mg/kg). Data are presented as means ± SEM. * *p* < 0.05; ** *p* < 0.01; *** *p* < 0.001; **** *p* < 0.0001 vs. control.

The L-arginine/ADMA ratio increased in both the PFC and hippocampus after acute MK-801 treatment (F_(2.26)_ = 12.22, *p* < 0.0001 and F_(2.27)_ = 7.6, *p* < 0.001, respectively) and after acute scopolamine administration (F_(2.28)_ = 9.26, *p* < 0.0008 and F_(2.27)_ = 14.94, *p* < 0.0001, respectively) (Figure 3).

The L-arginine/SDMA ratio decreased in the PFC (F_(2.26)_ = 8.52, *p* < 0.001) and hippocampus after acute MK-801 treatments (F_(2.26)_ = 20.41, *p* < 0.0001). Scopolamine had no influence on this parameter (Figure 3).

The L-arginine/NMMA ratio decreased in the hippocampus after acute MK-801 treatment (F_(2.27)_ = 5.57, *p* < 0.008). No significant changes in the L-arginine/NMMA ratio after acute administration of scopolamine were observed (Figure 3).

#### 2.2.3. The Levels of eNOS, nNOS, DDAH1, PMRT1 and PMRT5

**Figure 4:**
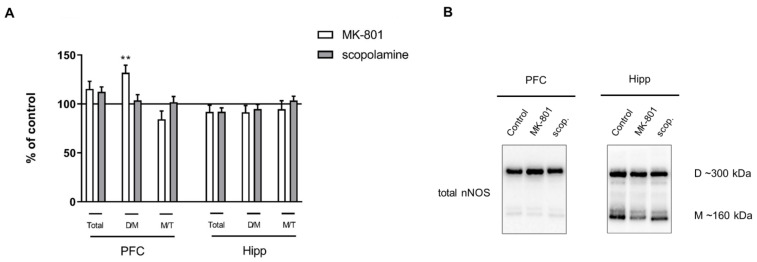
nNOS expression in the prefrontal cortex (PFC) and hippocampus (Hipp) after acute administration of MK-801 (0.3 mg/kg) or scopolamine (1 mg/kg) (**A**). Representative Western blots (**B**). D/M—dimer/monomer ratio; M/T—monomer/total ratio. Data are presented as means ± SEM. ** *p* < 0.01 vs. control.

Acute MK-801 administration increased the dimer/monomer (D/M) ratio of nNOS in the PFC (F_(2.27)_ = 5.169, *p* < 0.01), and no changes were observed in the total protein or the monomer/total protein (M/T) ratio. No significant changes in total nNOS or the M/T ratio were observed in either of the investigated structures after acute scopolamine administration (Figure 4).

In the PFC, acute MK-801 administration decreased the total eNOS protein (F_(2.26)_ = 6.36, *p* < 0.03). The M/T ratio was increased after acute MK-801 administration (F_(2.27)_ = 6.7, *p* < 0.03). After acute scopolamine administration, the total eNOS protein level decreased in the PFC (F_(2.26)_ = 6.36, *p* < 0.004). 

**Figure 5:**
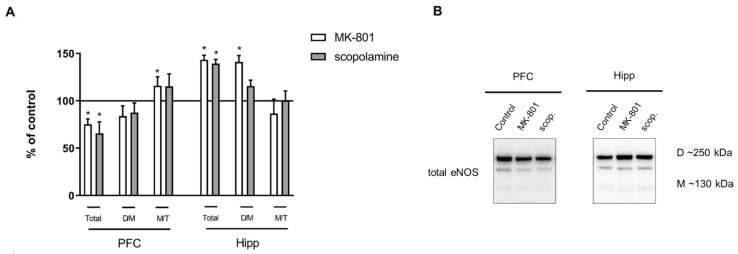
eNOS expression in the prefrontal cortex (PFC) and hippocampus (Hipp) after acute administration of MK-801 (0.3 mg/kg) or scopolamine (1 mg/kg) (**A**). Representative Western blots (**B**). D/M—dimer/monomer ratio; M/T—monomer/total ratio. Data are presented as means ± SEM. * *p* < 0.05 vs. control.

In the hippocampus, MK-801 induced an increase in total eNOS protein (F_(2.27)_ = 14.79, *p* < 0.0001) and D/M ratio (F_(2.26)_ = 14.63, *p* < 0.001), while scopolamine administration induced an increase in eNOS total protein (F_(2.25)_ = 14.07, *p* < 0.0003) (Figure 5).

**Figure 6:**
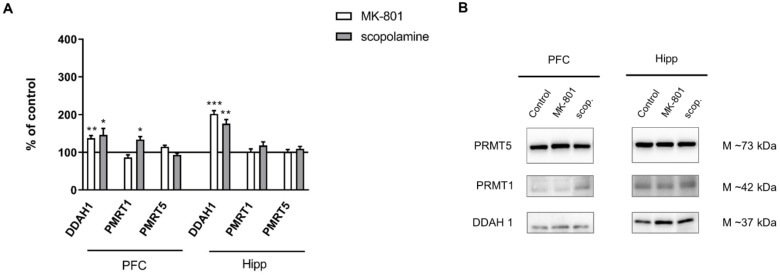
DDAH1, PMRT1 and PMRT5 expression in the prefrontal cortex (PFC) and hippocampus (Hipp) after acute administration of MK-801 (0.3 mg/kg) or scopolamine (1 mg/kg) (**A**). Representative Western blots (**B**). Data are presented as means ± SEM. * *p* < 0.05; ** *p* < 0.01; *** *p* < 0.001 vs. control.

DDAH1 expression increased in the PFC and in the hippocampus after acute MK-801 administration (F_(2.25)_ = 4.92, *p* < 0.01; F_(2.27)_ = 11.8, *p* < 0.0002, respectively). Acute scopolamine administration also induced an increase in DDAH1 expression both in the PFC and in the hippocampus (F_(2.27)_ = 6.7, *p* < 0.009 and F_(2.27)_ = 11.8, *p* < 0.003, respectively) (Figure 6).

No changes in the expression of PMRT1 or PMRT5 were observed after acute MK-801 treatment. The expression of PMRT1 increased only in the PFC after acute administration of scopolamine (F_(2.27)_ = 8.71, *p* < 0.0001). No changes in PMRT5 in either structure were observed after acute scopolamine treatment (Figure 6).

#### 2.2.4. cGMP and NMDA Receptor Expression

**Figure 7:**
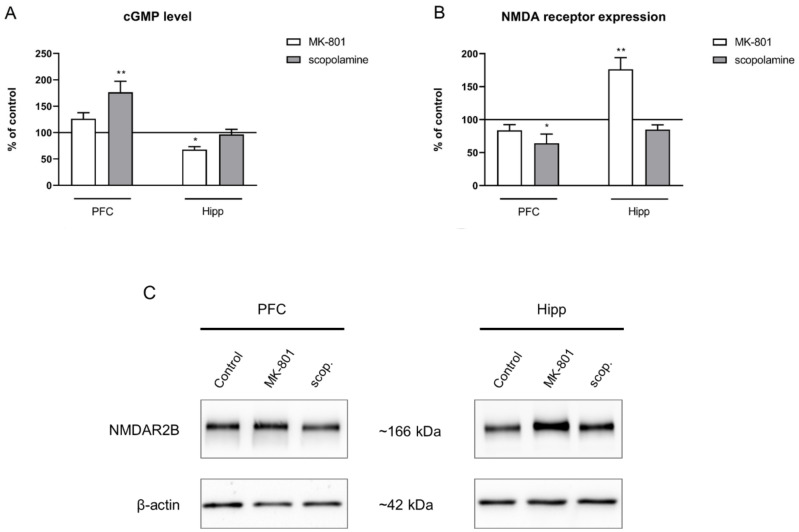
cGMP level (**A**) and GluN2B subunit-containing NMDA receptor expression (**B**) in the prefrontal cortex (PFC) and hippocampus (Hipp) after acute administration of MK-801 (0.3 mg/kg) or scopolamine (1 mg/kg). Representative Western blots (**C**). Data are presented as means ± SEM. * *p* < 0.05; ** *p* < 0.01 vs. control.

Acute MK-801 administration decreased the cGMP level only in the hippocampus (F_(2.24)_ = 5.38, *p* < 0.01). Acute scopolamine administration increased the cGMP level in the PFC (F_(2.27)_ = 5.53, *p* < 0.01) but not in the hippocampus (Figure 7A).

GluN2B subunit-containing NMDA receptor expression was not changed in the prefrontal cortex after MK-801 injection. In the hippocampus, acute MK-801 administration induced a significant increase in the expression of theGluN2B subunit-containing NMDA receptor (F_(2.24)_ = 7.51, *p* < 0.02). Acute scopolamine administration induced a significant decrease in the expression of the GluN2B subunit-containing NMDA receptor in the PFC (F_(2.24)_ = 5.55, *p* < 0.01) (Figure 7B).

### 2.3. Chronic MK-801 or Scopolamine Administration

#### 2.3.1. Amino Acids in PFC and Hippocampus

**Figure 8:**
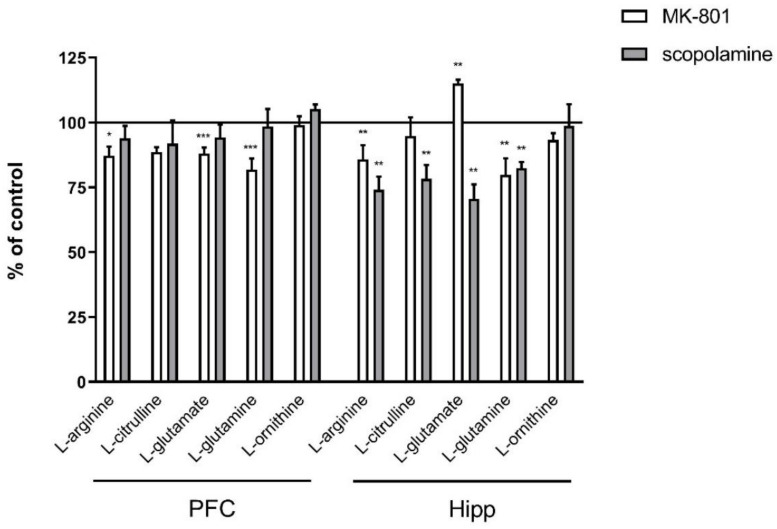
Amino acid levels in the prefrontal cortex (PFC) and hippocampus (Hipp) after chronic (14 days) administration of MK-801 (0.3 mg/kg) or scopolamine (1 mg/kg). Data are presented as means ± SEM. * *p* < 0.05; ** *p* < 0.01; *** *p* < 0.001 vs. control.

In the PFC, chronic administration of MK-801 induced significant decreases in L-arginine (F_(2.27)_ = 5.96; *p* < 0.007), L-glutamate (F_(2.25)_ = 17.51, *p* < 0.0001) and L-glutamine (F_(2.27)_ = 18.8, *p* < 0.0001). No changes in L-citrulline or L-ornithine levels were observed. Chronic scopolamine administration did not induce any significant changes in L-arginine, L-citrulline, L-glutamate, L-glutamine or L-ornithine levels (Figure 8).

In the hippocampus, chronic administration of MK-801 induced a significant decrease in L-arginine (F_(2.26)_ = 8.16; *p* < 0.001) and L-glutamine (F_(2.26)_ = 6.9, *p* < 0.003) and an increase in L-glutamate (F_(2.27)_ = 7.56, *p* < 0.002) levels. No changes in L-citrulline or L-ornithine levels were observed. After 14-day administration of scopolamine, decreases in L-arginine (F_(2.27)_ = 9.28, *p* < 0.0009), L-citrulline (F_(2.27)_ = 5.84, *p* < 0.007), L-glutamate (F_(2.27)_ = 15.59, *p* < 0.0001) and L-glutamine (F_(2.27)_ = 8.72, *p* < 0.001) were observed. No changes in L-ornithine level were detected (Figure 8).

#### 2.3.2. L-Arginine Derivatives in PFC and Hippocampus

**Figure 9:**
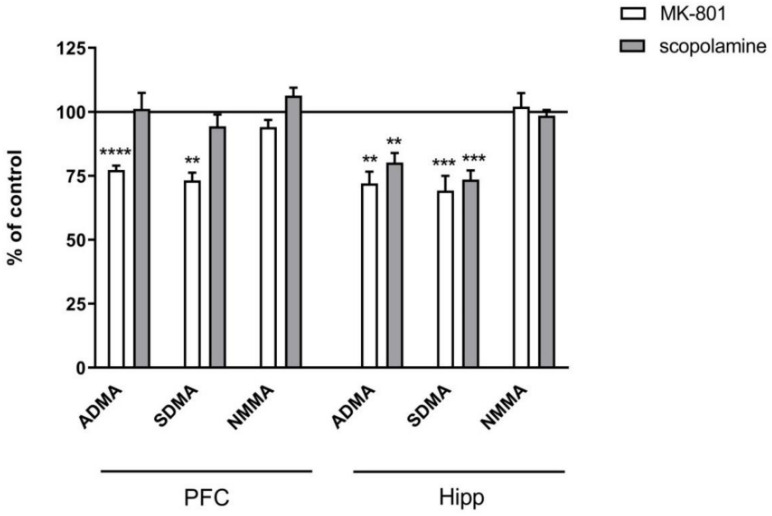
ADMA, SDMA and NMMA levels in the prefrontal cortex (PFC) and hippocampus (Hipp) after chronic (14 days) administration of MK-801 (0.3 mg/kg) or scopolamine (1 mg/kg). Data are presented as means ± SEM. ** *p* < 0.01; *** *p* < 0.001; **** *p* < 0.0001 vs. control.

In the PFC, chronic treatment with MK-801 induced a decrease in ADMA (F_(2.27)_ = 9.3, *p* < 0.0007) and SDMA (F_(2.27)_ = 12.26, *p* < 0.0001). No changes after chronic scopolamine treatment were noticed (Figure 9).

In the hippocampus, chronic MK-801 administration decreased ADMA (F_(2.33)_ = 12.74, *p* < 0.0001) and SDMA (F_(2.27)_ = 15.38, *p* < 0.0001). Chronic scopolamine administration induced decreases in ADMA (F_(2.25)_ =8.14, *p* < 0.001) and SDMA (F_(2.25)_ = 15.34, *p* < 0.0001). No changes in the NMMA level were detected (Figure 9).

**Figure 10:**
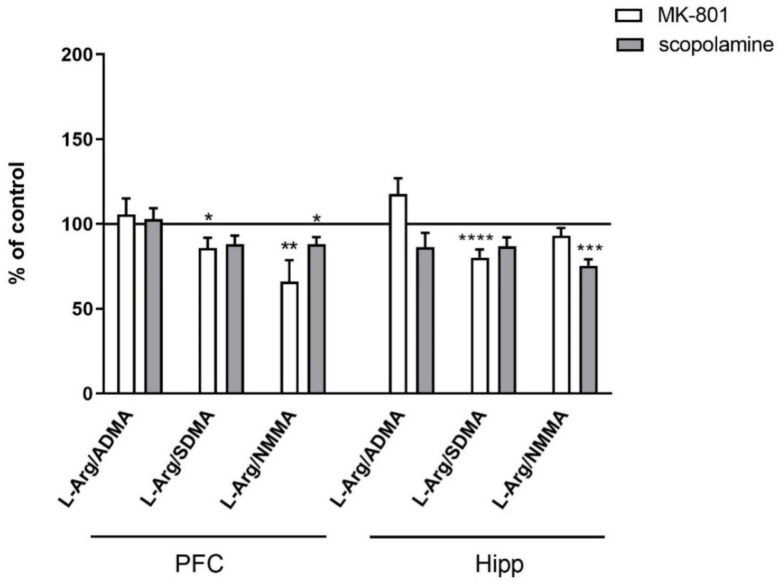
L-arginine (L-Arg)/ADMA, L-arginine/SDMA and L-arginine/NMMA ratios in the prefrontal cortex (PFC) and hippocampus (Hipp) after chronic (14 days) administration of MK-801 (0.3 mg/kg) or scopolamine (1 mg/kg). Data are presented as mean ± SEM. * *p* < 0.05; ** *p* < 0.01; *** *p* < 0.001; **** *p* < 0.0001 vs. control.

No changes in the L-arginine/ADMA ratio were observed after chronic MK-801 or scopolamine treatment (Figure 10).

The L-arginine/SDMA ratio decreased in the PFC (F_(2.26)_ = 8.52, *p* < 0.001) and hippocampus (F_(2.26)_ = 20.41, *p* < 0.0001) after chronic MK-801 treatments. The L-arginine/SDMA ratio was not changed in any of the investigated structures after chronic scopolamine administration (Figure 10).

The L-arginine/NMMA ratio decreased in the PFC after repeated MK-801 treatment (F_(2.27)_ = 9.91, *p* < 0.0006). Scopolamine administration decreased the L-arginine/NMMA ratio in the PFC (F_(2.27)_ = 8.22, *p* < 0.001) and in the hippocampus (F_(2.27)_ = 13.89, *p* < 0.0001) (Figure 10).

#### 2.3.3. The Levels of nNOS, eNOS, DDAH1, PMRT1 and PMRT5

**Figure 11:**
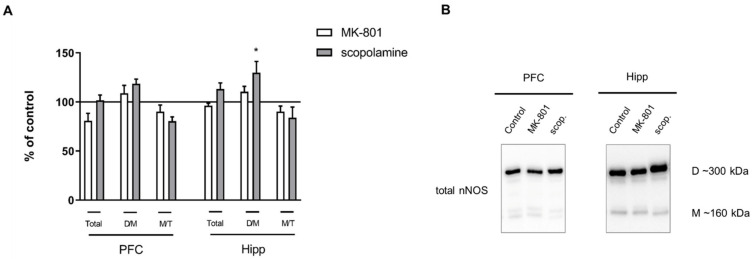
nNOS expression in the prefrontal cortex (PFC) and hippocampus (Hipp) after chronic (14 days) administration of MK-801 (0.3 mg/kg) or scopolamine (1 mg/kg) (**A**). Representative Western blots (**B**). D/M—dimer/monomer ratio; M/T—monomer/total ratio. Data are presented as means ± SEM. * *p* < 0.05 vs. control.

No significant changes in total nNOS or the M/T ratio were observed in either of the investigated structures after chronic MK-801 or scopolamine administration. Chronic scopolamine treatment induced a slight increase in the nNOS D/M ratio, which reached significance in the hippocampus (F_(2.26)_ = 3.5, *p* < 0.04) (Figure 11).

**Figure 12:**
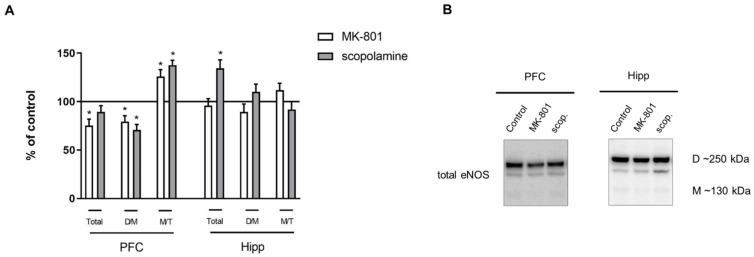
eNOS expression in the prefrontal cortex (PFC) and hippocampus (Hipp) after chronic (14 days) administration of MK-801 (0.3 mg/kg) or scopolamine (1 mg/kg) (**A**). Representative Western blots (**B**). D/M—dimer/monomer ratio; M/T—monomer/total ratio. Data are presented as means ± SEM. * *p* < 0.01 vs. control.

Chronic MK-801 administration decreased the total eNOS protein (F_(2.26)_ = 6.36, *p* < 0.03) and D/M ratio (F_(2.26)_ = 14.63, *p* < 0.001) in the PFC. The M/T ratio was increased (F_(2.27)_ = 6.7, *p* < 0.03). Chronic scopolamine treatment induced a decrease in the eNOS D/M ratio (F_(2.26)_ = 14.63, *p* < 0.001) and an increase in the M/T ratio (F_(2.27)_ = 6.7, *p* < 0.002) in the PFC (Figure 12).

In the hippocampus, MK-801 induced no changes, while chronic scopolamine treatment induced an increase only in total eNOS protein (F_(2.26)_ = 5.8, *p* < 0.02) (Figure 12).

**Figure 13:**
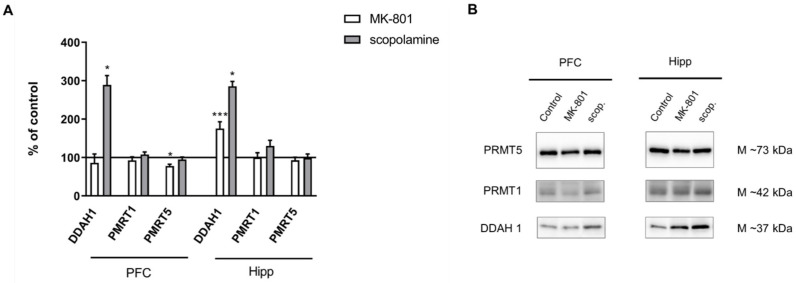
DDAH1, PMRT1 and PMRT5 expression in the prefrontal cortex (PFC) and hippocampus (Hipp) after chronic (14 days) administration of MK-801 (0.3 mg/kg) or scopolamine (1 mg/kg) (**A**). Representative Western blots (**B**). Data are presented as means ± SEM. * *p* < 0.05; *** *p* < 0.001 vs. control.

DDAH1 expression increased in the hippocampus after chronic MK-801 administration (F_(2.27)_ = 10.09, *p* < 0.0005). Chronic scopolamine administration induced an increase in DDAH1 expression both in the PFC and in the hippocampus (F_(2.27)_ = 6.7, *p* < 0.009 and F_(2.27)_ = 11.8, *p* < 0.003, respectively). Neither MK-801 nor scopolamine treatment influenced the level of PMRT1. The expression of PMRT5 decreased in the PFC after chronic MK-801 administration (F_(2.26)_ = 3.6, *p* < 0.03) (Figure 13).

#### 2.3.4. cGMP and NMDA Receptor Expression

**Figure 14:**
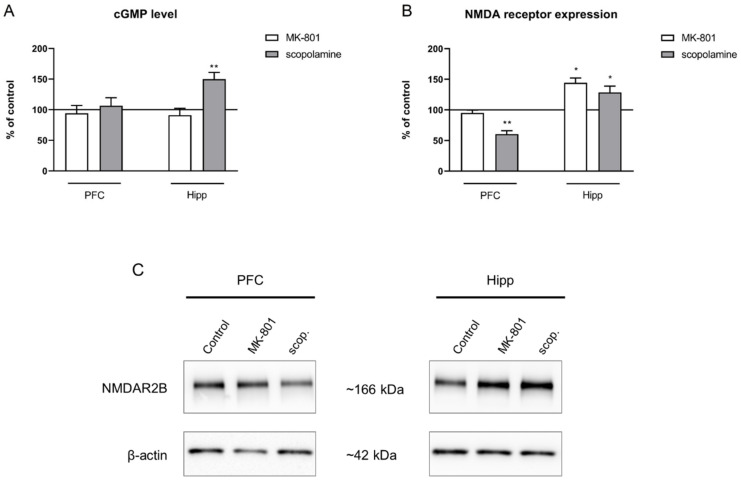
cGMP level (**A**) and GluN2B subunit-containing NMDA receptor expression (**B**) in the prefrontal cortex (PFC) and hippocampus (Hipp) after chronic (14 days) administration of MK-801 (0.3 mg/kg) or scopolamine (1 mg/kg). (**C**) Representative Western blots. Data are presented as means ± SEM. * *p* < 0.05; ** *p* < 0.01 vs. control.

No changes after chronic MK-801 administration were observed in the cGMP level in either of the investigated structures. Chronic scopolamine administration increased the cGMP level in the hippocampus (F_(2.28)_ = 8.91, *p* < 0.001), with no impact on the cGMP level in the PFC (Figure 14A).

GluN2B subunit-containing NMDA receptor expression was not changed in the prefrontal cortex after MK-801 administration. In the hippocampus, chronic MK-801 administration induced a significant increase in the expression of theGluN2B subunit-containing NMDA receptor (F_(2.24)_ = 7.51, *p* < 0.02). Chronic scopolamine administration induced a significant decrease in the expression of the GluN2B subunit-containing NMDA receptor in the PFC (F_(2.24)_ = 5.55, *p* < 0.01). An increase in GluN2B subunit-containing NMDA receptor expression in the hippocampus was observed after chronic administration of scopolamine (F_(2.27)_ = 5.4, *p* < 0.01) (Figure 14B).

### 2.4. Amino Acid Levels and L-Arginine Derivatives in Plasma after MK-801 and Scopolamine Administration

#### 2.4.1. Acute Administration

Of the analyzed amino acids, L-glutamine (427.2 µM) had the highest concentration in plasma, followed by L-citrulline (67.8 µM) and L-ornithine (65.9 µM). L-arginine (44.4 µM) and L-glutamate (44.19 µM) had the lowest concentrations.

**Figure 15:**
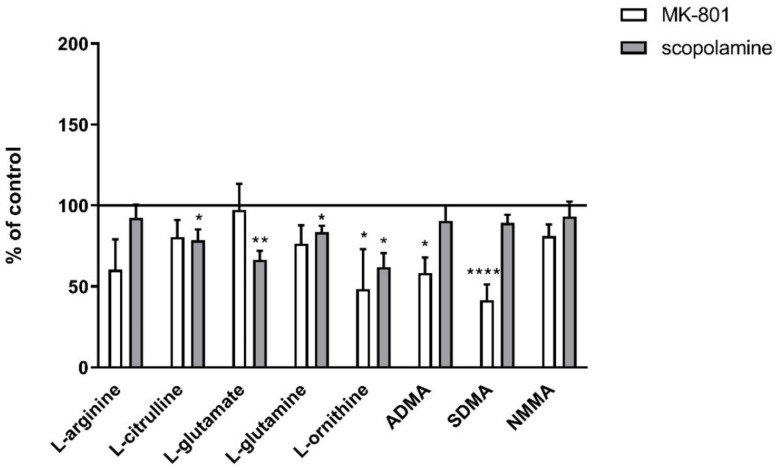
Amino acid and L-arginine derivative levels in plasma after acute administration of MK-801 (0.3 mg/kg) or scopolamine (1 mg/kg). Data are presented as means ± SEM. * *p* < 0.05; ** *p* < 0.01; **** *p* < 0.0001 vs. control.

Acute administration of MK-801 induced a significant decrease in L-ornithine (F_(2.25)_ = 5.59, *p* < 0.007). Acute administration of scopolamine induced a significant decrease in L-citrulline (F_(2.26)_ = 4.22, *p* < 0.02), L-glutamate (F_(2.26)_ = 6.12, *p* < 0.006), L-glutamine (F_(2.26)_ = 4.06, *p* < 0.02) and L-ornithine (F_(2.26)_ = 7.19, *p* < 0.003) (Figure 15).

In the plasma of control animals, ADMA constituted ca. 64% (0.79 µM) of L-arginine derivatives, followed by NMMA at 27% (0.33 µM) and SDMA at 9% (0.11 µM). Acute administration of MK-801 decreased ADMA (F_(2.34)_ = 4.71, *p* < 0.01) and SDMA (F_(2.34)_ = 11.6, *p* < 0.0001). No changes in L-arginine derivatives were observed after scopolamine administration (Figure 15).

**Figure 16:**
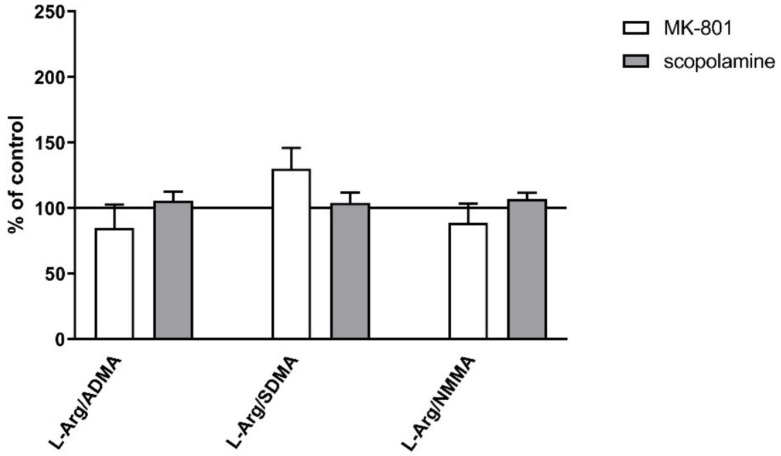
L-arginine (L-Arg)/ADMA, L-arginine/SDMA and L-arginine/NMMA ratios in plasma after acute administration of MK-801 or scopolamine. Data are presented as means ± SEM.

No changes were observed in plasma after acute administration of MK-801 01 or scopolamine (Figure 16).

#### 2.4.2. Chronic Administration

**Figure 17:**
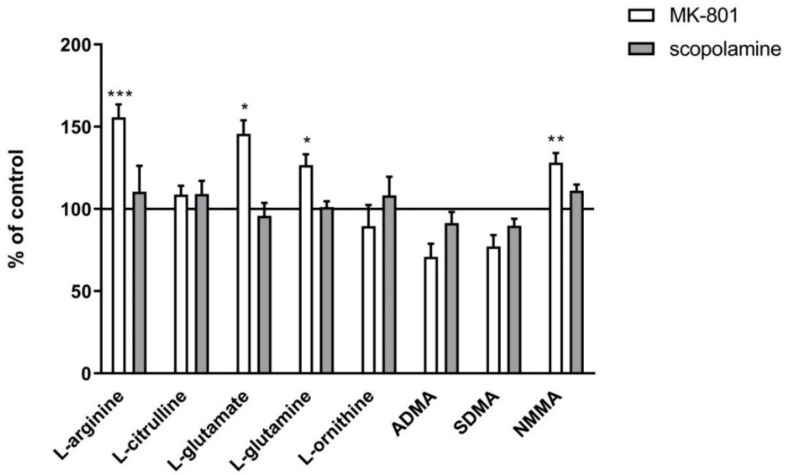
Amino acid and L-arginine derivative levels in plasma after chronic (14 days) administration of MK-801 (0.3 mg/kg) or scopolamine (1 mg/kg). Data are presented as means ± SEM. * *p* < 0.05; ** *p* < 0.01; *** *p* < 0.001 vs. control.

In plasma, chronic MK-801 treatment induced an increase in L-arginine (F_(2.28)_ = 14.44, *p* < 0.001), L-glutamate (F_(2.28)_ = 4.94, *p* < 0.01) and L-glutamine (F_(2.28)_ = 8.49, *p* < 0.0009). No changes in L-citrulline levels were observed. No changes after chronic scopolamine treatment were observed (Figure 17).

**Figure 18:**
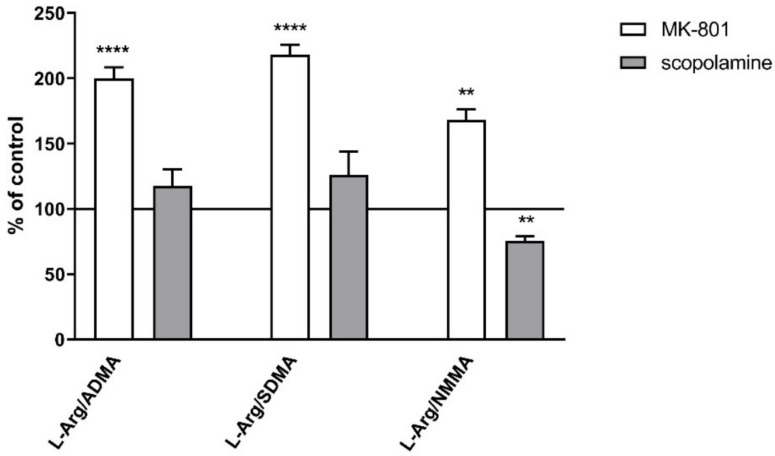
L-arginine (L-Arg)/ADMA, L-arginine/SDMA and L-arginine/NMMA ratios in plasma after chronic (14 days) administration of MK-801 (0.3 mg/kg) or scopolamine (1 mg/kg). Data are presented as means ± SEM. ** *p* < 0.01; **** *p* < 0.0001 vs. control.

Chronic treatment with MK-801 increased L-arginine/ADMA, L-arginine/SDMA and L-arginine/NMMA ratios (F_(2.26)_ = 19.05, *p* < 0.0001, F_(2.26)_ = 13.89, *p* < 0.0001 and F_(2.26)_ = 9.42, *p* < 0.0008, respectively). Chronic administration of scopolamine decreased the L-arginine/NMMA ratio (F_(2.27)_ =13.89, *p* < 0.0001) (Figure 18).

### 2.5. S-Nitrosylation

**Figure 19:**
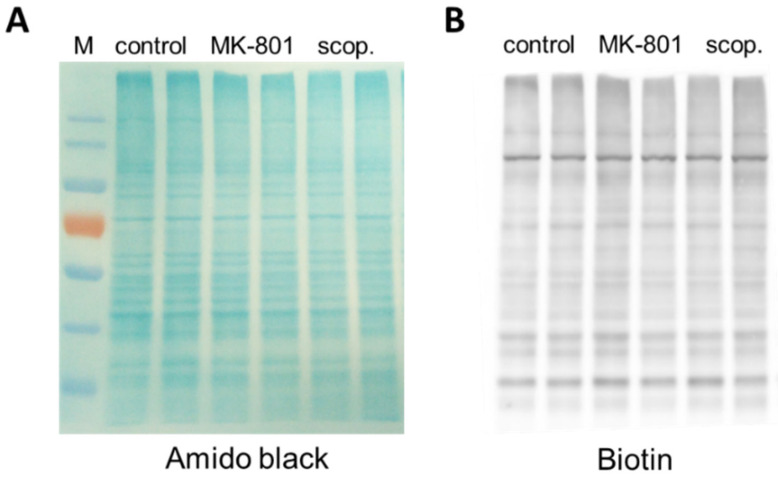
Representative results demonstrating the effects of chronic (14 days) administration of MK-801 or scopolamine on protein S-nitrosylation of the prefrontal cortex samples. (**A**) Proteins visualized by Amido Black staining on nitrocellulose membrane, (**B**) Western blot of total nitrosylated proteins labeled in biotin switch assay on the same membrane. M—protein marker.

Before estimating the S-nitrosylation of individual proteins, each sample was separated in polyacrylamide gel, and total S-nitrosylation after treatment with MK-801 or scopolamine was compared to that in the control group. There were no significant differences between the groups (Figure 19).

**Figure 20:**
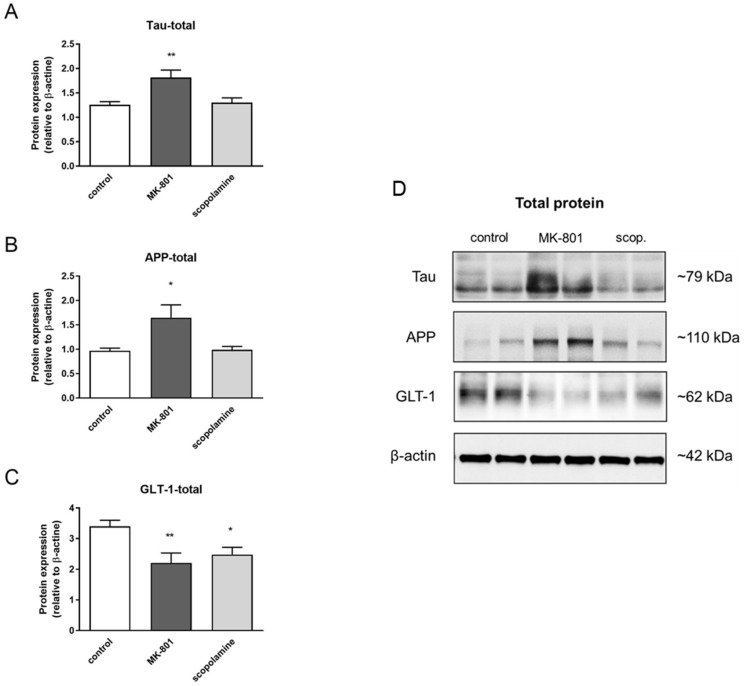
Densitometric analysis of Western blot results demonstrating changes in the expression of Alzheimer precursor protein (APP), tau and glutamate transporter 1 (GLT-1) in the prefrontal cortex after chronic (14 days) administration of MK-801 (0.3 mg/kg) or scopolamine (1 mg/kg) (**A**–**C**). Expression of total protein was normalized to β-actin. Representative Western blots (**D**). Data are presented as means ± SEM. * *p* < 0.05; ** *p* < 0.01 compared to the untreated control group.

The total expression patterns of 5 proteins after MK-801 or scopolamine administration over 14 days relative to β-actin were evaluated. The upregulation of the expression of tau and Alzheimer precursor protein (APP) was statistically significant after MK-801 treatment (F_(2.24)_ = 7.21, *p* < 0.003 and F_(2.29)_ = 5.88, *p* < 0.007, respectively) (Figure 20A,B). Additionally, chronic administration of MK-801 and scopolamine resulted in a statistically significant reduction in the expression of glutamate transporter 1 (GLT-1) relative to the control (F_(2.25)_ = 6, *p* < 0.008 and F_(2.23)_ = 5.88, *p* < 0.009, respectively) (Figure 20C). The level of downregulation was similar in both groups.

**Figure 21:**
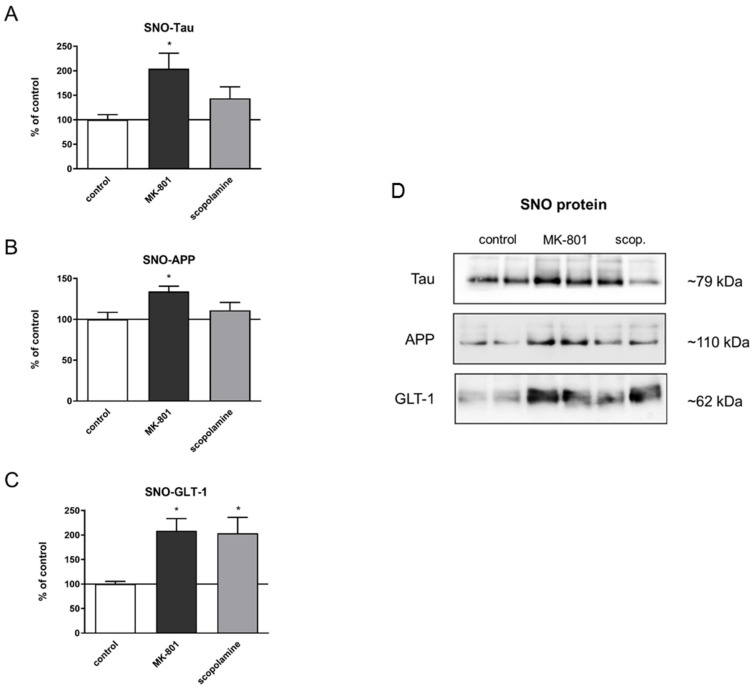
Densitometric analysis of SNO-proteins (**A**–**C**), equal volumes of eluates from the streptavidin resin were loaded into acrylamide gels. Representative Western blots (**D**). Data are presented as means ± SEM. * *p* < 0.05 compared to the untreated control group.

Comparison between the total expression of tau or APP protein and their S-nitrosylated forms showed very similar patterns (F_(2.24)_ = 4.95, *p* < 0.001; F_(2.23)_ = 4.45, *p* < 0.02, respectively) (Figure 21A,B). Chronic administration of MK-801 and scopolamine significantly upregulated the S-nitrosylation of GLT-1 compared to the control group (F_(2.23)_ = 6.65, *p* < 0.005) (Figure 20C).

## 3. Discussion

In the present study, acute and chronic administration of MK-801 (0.3 mg/kg) or scopolamine (1 mg/kg) led to alterations in NO-dependent pathways that may contribute to the cognitive decline induced by these compounds and, to some extent, resemble neurochemical changes observed in humans.

Acute MK-801 injection induced decreases in L-arginine, L-glutamate and L-glutamine levels both in the PFCs and hippocampi, and the effect was observed in the washout period after chronic treatment, accompanied by significant and persistent decreases in L-Arg/SDMA and L-Arg/NMMA ratios. Contrary to expectations, a transient increase (observed after acute administration only) in the L-Arg/ADMA ratio was detected. There are neither clinical nor preclinical reports concerning ADMA in schizophrenia or other CNS disorders, but increased ADMA in relation to L-arginine has been reported to contribute to cardiovascular pathologies [23]. This result may be irrelevant to present considerations, as the obtained results indicate that SDMA plays a prominent role in the brain (60–65% compared to ADMA/NMMA) and are in line with a report indicating that the overall ADMA content was lowest in the brain compared to other tissues [24]. Additionally, upon the administration of MK-801, a significant increase in DDAH1 expression was observed (the enzyme plays a critical role in ADMA degradation) [25]; therefore, the rapid metabolism of ADMA (a putative compensatory mechanism) after MK-801 treatment cannot be excluded. Hence, SDMA and NMMA together constitute ~70% of methylamines in the brain, where they interfere with the production of NO from L-arginine, and imbalanced proportions that are unfavorable for L-arginine after MK-801 treatment indicate subsequent insufficient NO production by constitutive NO synthases (cNOS) [23,26,27].

Contrary to expectations, MK-801 administration had an insignificant effect on nNOS expression, a crucial enzyme in neuronal NO production, subsequent NMDA receptor activation and cGMP synthesis, which is believed to mediate long-term potentiation (LTP) and memory consolidation [28,29,30]. In contrast to expectations, the nNOS D/M ratio increased in the PFC. Since proper NO production is facilitated by dimers, in contrast to monomers, which trigger oxidative stress through the generation of reactive oxygen/reactive nitrogen species (ROS/RNS) [31,32], the impact of MK-801 on nNOS is not detrimental. The effect was not persistent and not observed in the washout period, and the expression of total nNOS protein and monomer content remained unchanged.

Concomitant experiments aimed to assess the main factors mediating NO-induced effects, i.e., the cGMP level accompanied by NMDA receptor expression, and the results reveal that cGMP production was abolished in the hippocampus with increased NMDA receptor expression, indicating the attenuation of cGMP synthesis in the MK-801-based model, followed by the diminution of LTP.

Contrary to its impact on nNOS, MK-801 induced significant eNOS dysfunction, decreasing its level in the PFC and hippocampus, accompanied by increased monomer content, which may elevate the risk of generating oxidative stress. The increase in the D/M ratio observed shortly after the administration of MK-801 in the hippocampus might suggest the rapid decomposition of dimers into monomers.

In contrast to MK-801, the administration of scopolamine decreased amino acid levels (except L-ornithine), predominantly in the hippocampal formation, accompanied by reduced quantities of ADMA and SDMA in both structures. The observed decrease in the L-Arg/NMMA ratio as a consequence of chronic scopolamine administration may indicate diminished L-arginine availability for NO production. The simultaneous increase in the DDAH1 level could indicate the rapid elimination of ADMA, as mentioned above when discussing the MK-801-induced effect.

No significant changes in nNOS expression, except an increase in the D/M ratio in the hippocampal formation after 14 days of treatment, were detected. The effect was comparable to that observed after MK-801. Treatment with scopolamine had a less detrimental effect on eNOS activity and largely affected the PFC, in which an increased level of monomers was observed.

In contrast to MK-801, scopolamine treatment increased the level of cGMP both in the PFC and in the hippocampus, in which an increase was also observed in the washout period. As the expression of NMDA receptors was decreased, the elevation of the cGMP level could not be the result of the stimulation of the NMDA receptor.

Our results showing no or slight changes in nNOS expression are not as expected, mainly because both MK-801 and scopolamine are proven to impair LTP in rodents [33,34,35]. A rare animal study indicates decreased NOS activity after scopolamine administration in the hippocampal region of the dentate gyrus only, with no changes in nNOS/eNOS expression [36]. However, no complex studies presenting the impact of single and chronic administration of MK-801 or scopolamine on nNOS, eNOS and dimer/monomer ratios are available. Numerous clinical data describe alterations in the cellular expression of brain-associated NOS in schizophrenia or AD. Remarkably, both decreases and increases in NOS activity, NOS protein and/or mRNA content have been found in schizophrenia [37] andAD [38,39].

MK-801 induces its effects viaa mechanism that involves glutamatergic hyperactivity in the brain and is associated with the deficiency of NMDA receptors located on inhibitory GABAergic interneurons (described as the glutamatergic theory of schizophrenia in [40,41,42]). On the other hand, acetylcholine projections in hippocampal neurons mediating LTP are inhibited by scopolamine [43,44]. NO-mediated synaptic plasticity involves not only LTP but also long-term depression (LTD) [45,46,47], and both processes can be induced not only in glutamatergic but also in GABAergic synapses [48] and can be inhibited by MK-801 [49] or scopolamine [50]. Approximately 60% of neurons are glutamatergic; however, the remaining 40% of GABAergic neurons use nNOS as well. It is impossible to precisely determine neuron-specific changes in nNOS expression by Western blotting. We hypothesize that MK-801 or scopolamine may shift the balance between LTP and LTD, thus contributing to memory impairment, without affecting the absolute pool of nNOS in the brain homogenate. The nNOS level may increase as a consequence of an astrocytic response to MK-801 or scopolamine treatment, but this requires further studies.

While the neuronal pool of NO is dependent on the activity of nNOS, eNOS catalyzes the production of NO in the epithelium and is essential for a healthy cardiovascular system [51,52]. In the CNS, eNOS ensures proper cerebral blood flow and is crucial for the integrity of the blood–brain barrier (BBB) [53,54]. eNOS deficiency was described as the principal factor contributing to neurodegenerative processes, including AD, predominantly by facilitating the cerebral accumulation of amyloid-β peptides (Aβ) and the microtubule-associated protein tau, leading to the formation of extracellular amyloid plaques and intracellular neurofibrillary tangles [55,56]. eNOS deficiency was observed in animal models of AD with the use of KO animals [57].

Less is known about the role of eNOS in schizophrenia, and data concerning eNOS expression in animal models of schizophrenia are lacking. However, it has been reported that BBB breakdown is associated with cognitive decline in both animals and humans [58,59], and the complex nature of BBB dysfunction in psychosis might be relevant to many aspects of disrupted neuronal and synaptic function, increased permeability to inflammatory molecules and disrupted glutamate homeostasis [60]. The present results might indicate that eNOS deficiency and the putative subsequent impairment of BBB permeability may constitute one of the causes underlying MK-801-induced cognitive decline.

Aberrant NO-mediated S-nitrosylation processes have been shown to be responsible for Aβ cleavage, tau phosphorylation [61] and dysfunction of the activity of GLT-1 transport protein, which is responsible for glutamate reuptake, a crucial process in both AD and schizophrenia pathology [62,63]. Our results are the first to show that the tool compounds MK-801 and scopolamine may induce their effects by promoting the S-nitrosylation of proteins that govern glutamate transport and metabolism (GLT-1). Additionally, administration of MK-801 elevated the expression of APP and tau proteins, but neither of the tool compounds induced any changes in their S-nitrosylation.

Other results obtained in the present study confirm that the glutamate–glutamine cycle, a process enabling metabolic trafficking between astrocytes and neurons [64,65], may be impaired due to MK-801 or scopolamine administration, as the levels of both amino acids were decreased, and the impact of MK-801 was evident in both structures. As L-citrulline or L-ornithine levels remained unchanged, it seems that neither MK-801 nor scopolamine affected the urea cycle [66].

The levels of amino acids and L-arginine derivatives were additionally determined in the plasma of MK-801- and scopolamine-treated mice. Scopolamine had less of an impact than MK-801 on amino acids and L-arginine derivatives in plasma, and the MK-801-induced effect was more beneficial than detrimental, as increases in amino acids followed by increases in L-Arg/methyloarginine ratios were detected, suggesting improved NO availability in plasma. The results are in contrast to clinical reports showing increased ADMA levels in the plasma of schizophrenic [67] and AD patients [68,69]. Some reports concerning the ADMA level in cerebrospinal fluid do not show an association between the ADMA level and the development of illness [70]. Plasma measurements were taken to provide a complete picture of drug effects but are not important for establishing the mechanisms of memory impairments.

## 4. Materials and Methods

### 4.1. Animals

Male Albino Swiss (20–25 g) mice (Charles River Laboratory, Sulzfeld, Germany) were used in all of the experiments. Male CD1 mice were used in all our previous studies related to cognitive impairment investigations and are generally used by others. All animals were kept under a 12:12 light–dark cycle at a room temperature of 22 ± 1 °C with free access to food and water. Each experimental group consisted of 10 animals. The compounds were administered at a volume of 10 mg/mL. Animal facility conditions were in accordance with EU Directive 2010/63/EU and subsequent ordinances of the Polish Ministry of Agriculture and Rural Development.The procedures were approved by the II Local Ethics Committee by the Maj Institute of Pharmacology, Polish Academy of Sciences in Krakow (65/2020).

### 4.2. Drugs

Scopolamine hydrobromide ((α,*S*)-α-(Hydroxymethyl)benzeneacetic acid (1α,2β,4β,5α,7β)-9-methyl-3-oxa-9-azatricyclo[3.3.1.02,4]non-7-yl ester hydrobromide), LY487379 (2,2,2-Trifluoro-*N*-[4-(2-methoxyphenoxy)phenyl]-*N*-(3-pyridinylmethyl) ethanesulfonamide hydrochloride) and MK-801 (5*S*,10*R*)-(+)-5-Methyl-10,11-dihydro-5*H*-dibenzo[*a*,*d*]cyclohepten-5,10-imine maleate)were obtained from Tocris (Bristiol, United Kingdom) and dissolved in saline.

The compounds were administered at doses of 1 mg/kg (scopolamine) or 0.3 mg/kg (MK-801). Animals were killed 30 min after acute administration or after 14 days of the treatments, 24 h after the last administration. Brain structures (hippocampi and prefrontal cortices) and plasma were collected for further analysis.

### 4.3. L-Arginine Levels and Its Derivatives

The plasma extraction (50 μL) was performed using a protein precipitation method, using three volumes of ice-cold acetonitrile containing an internal standard (IS) (L-Arg-C13, 4 µM). The extract was vortexed in a thermomixer for 15 min at 4 °C and followed by centrifugation at 14,000 rpm for 20 min at 4 °C. The supernatant was transferred to a new Eppendorf tube and evaporated to dryness in a vacuum evaporator at 45 °C for 1 h. Samples were reconstituted (50 μL) in 0.1%FA in water, then centrifuged at 14,000 rpm for 15 min at 4 °C and analyzed by means of LC/MS/MS for ADMA, SDMA and NMMA. A total of 5 μL of each supernatant was diluted to 50 μL with 0.1% FA in water containing 4 µM IS and analyzed for L-Arg, L-Cit, L-Glu, L-Gln and L-Orn. A QTRAP 4500 (AB Sciex, Framingham, MA, USA) mass spectrometer in positive ion mode in Multiple Reaction Monitoring (MRM screening) coupled with UHPLC (NEXERA XR, Shimadzu) was used. Chromatographic separation was achieved on anIntrada Amino Acid 3u 50 × 2 mm column (Imtakt, Portland, OR, USA) at a flow rate of 0.3 mL/min at 50 °C. The gradient program was as follows: 15–100% B (3 min), 100% B (0.5 min), 100–15% B (0.1 min), 15% B (3.4 min), solvent A (84% acetonitrile with 0.3% formic acid in 25 mM ammonium formate) and solvent B (20% acetonitrile in 200 mM ammonium formate). The Analyst software was used to control the instrument and collect data, and Multiquant 3.0.2 software was used for quantification analysis. The electrospray ionization source was fitted with a stainless-steel capillary (100 μm i.d.). The ion transfer tube temperature was 300 °C. The spray voltage, collision energy, declustering potential and gas parameters were optimized to achieve the maximum response using the test compounds. Selected reaction monitoring was carried out using the transition parameters presented in Table 1. The calibration curves were constructed, and a linear response was obtained in the ranges of 0.05–10 µM (ADMA, SDMA, NMMA), 3–100 µM (L-Arg, L-Cit), 6–200 µM (L-Glu, L-Orn) and 0.1–1000 µM (L-Gln) by spiking known amounts of each compound in control plasma.

Brain samples were ground to powder in liquid nitrogen, and suspended in a solution of 10% perchloric acid and acetonitrile (1:1 ratio) in a ratio of 200 μL buffer per 50 mg tissue. Samples were incubated in a thermomixer at 4 °C for 15 min and centrifuged at 12,000× *g* at 4 °C for 15 min. Samples were kept frozen at −80 °C for LC/MS/MS analysis.

For all compounds, two transition ions were selected: prime (in bold) was used for quantification, and the second was used for confirmation.

### 4.4. Western Blotting

Brain samples were ground to powder in liquid nitrogen, suspended in ice-cold RIPA lysis buffer (Cell Signaling Technology, Leiden, The Netherlands, Cat# 9806S) supplemented with PMSF (1 mM) and Protease Inhibitor Cocktail (Sigma-Aldrich, Darmstadt, Germany, Cat# P8340), and incubated on ice for 15 min. After incubation, the samples were centrifuged at 12,000× *g* at 4 °C for 15 min, the supernatants were harvested and the protein concentration was measured with the DC™ Protein Assay Kit II (Bio-Rad, Basel, Switzerland, Cat# 5000112).

A total of 40 µg of protein from each sample was diluted with Laemmli buffer (Bio-Rad, Basel, Switzerland, Cat# 1610747) containing 2.5% β-mercaptoethanol to a final volume of 10 µL. The samples were loaded on prechilled (4 °C) 4–15% polyacrylamide gels (Bio-Rad, Basel, Switzerland, Cat# 4568086) submerged in ice-cold running buffer (Bio-Rad, Basel, Switzerland, Cat# 1610772). Low-temperature SDS-PAGE was performed in an ice bath to ensure that the temperature of the gel remained at 4 °C.

After SDS-PAGE, the gels were visualized under UV light to measure total protein levels, and then the samples were transferred (Trans-Blot^®^ Turbo™ Transfer System, Bio-Rad, Basel, Switzerland) to PVDF membranes (Bio-Rad, Basel, Switzerland, Cat# 17001919). The membranes were blocked for 30 min with 5% BSA (Sigma-Aldrich, Darmstadt, Germany, Cat# A4503) for the detection of eNOS, nNOS, DDAH, PRMT5, PRMT1 and alpha 1 sodium potassium ATPase or with 1% blocking solution (BM Chemiluminescence Western Blotting Kit (Mouse/Rabbit), Roche, Sigma-Aldrich, Darmstadt, Germany, Cat# 11520709001) for detection of NMDAR2B and β-actin. Overnight incubation at 4 °C was performed with the following primary rabbit monoclonal antibodies: anti-eNOS (1:1000 dilution, Cell Signaling Technology, Leiden, The Netherlands, Cat# 32027S), anti-nNOS (1:1000 dilution, Cell Signaling Technology, Leiden, The Netherlands, Cat# 4231S), anti-DDAH1 (1:10,000 dilution, Abcam, Cambridge, UK, Cat# ab180599), anti-PRMT5 (1:10,000 dilution, Abcam, Cat# ab109451), anti-PRMT1 (1:1000 dilution, Abcam, Cambridge, UK, Cat# ab190892) or rabbit monoclonal antibody anti-NMDAR2B (1:1000 dilution, Abcam, Cambridge, UK, Cat# ab183942) and mouse monoclonal antibody anti-β-actin (1:7500 dilution, Sigma Aldrich, Darmstadt, Germany, Cat# A5441). Incubation with HRP anti-alpha 1 sodium potassium ATPase (1:5000 dilution, Abcam, Cambridge, UK, Cat# ab196696) was performed for 1 h at room temperature. The next day, the membranes were washed extensively with TBS-T and incubated for 1 h at room temperature with a goat anti-rabbit IgG HRP-conjugated secondary antibody (1:2000 dilution, Cell Signaling Technology, Leiden, The Netherlands, Cat# 7074S) or anti-mouse IgG-peroxidase conjugated/anti-rabbit IgG-peroxidase conjugated antibody (BM Chemiluminescence Western Blotting Kit, 1:7000) (NMDA and β-actin), washed again with TBS-T and developed using VisiGlo™ HRP chemiluminescentsubstrate (VWR, Radnor, PA, USA, Cat# 1B1583KIT) or BM Chemiluminescence Western Blotting Kit (NMDA and β-actin). To measure eNOS, nNOS, DDAH1, PRMT5, PRMT1, alpha 1 sodium potassium ATPaseprotein, NMDAR2B and β-actin expression, densitometry was performed using Image Lab software v. 4.1 (BioRad, Basel, Switzerland). Alpha 1 sodium potassium ATPase or β-actinexpression levels were used for normalization. The density of the obtained bands of the studied proteins was first normalized to alpha 1 sodium potassium ATPase bands, and then the ratio was calculated.

eNOS and nNOS levels were analyzed as total protein, dimer/monomer (D/M) ratio and monomer/total protein (M/T) ratio [71].

### 4.5. Detection of Protein S-Nitrosylation Using Biotin Switch Assay

The post-transcriptional modification of cysteine by NO was performed according to Forrester et al. [72]. Briefly, prefrontal cortices were homogenized in 1.8 mL of lysis buffer (25 mM HEPES, 50 mM NaCl, 0.1 mM 1% NP-40, 0.5 mM PMSF + protease inhibitors, pH 7.4) using TissueLizer II from Qiagen (Hilden, Germany) (25 Hz; 3 min, stainless steel beads 5 mm). After centrifugation (2000× *g*; 10 min), protein concentration was measured by the BCA method, and 1.5 mg of homogenate was further processed. To 1.8 mL of homogenate in HEN buffer, 200 µL of SDS 25% and 20 µL of MMTS 20% were added. Samples were incubated at 50 °C in the dark for 20 min and then precipitated in acetone. The protein pellets were resuspended in 240 µL of HENS buffer (100 mM HEPES, 1 mM EDTA, 0.1 mM neocuproine pH 8.0, 1% SDS) and labeled by means of biotin-HPDP (Sigma-Aldrich, Darmstadt, Germany) in the presence of 80 mM of sodium ascorbate (RT, in the dark for 60 min). After precipitation in acetone, the S-nitrosylated proteins were pulled down after overnight incubation at 4 °C in the presence of avidin affinity resin. The samples were eluted in 100 µL of HENS (10 min at 95 °C). After centrifugation (5000× *g*; 30 s), supernatants were collected and mixed with 4× non-reducing Laemmli loading buffer. The S-nitrosylation of proteins was evaluated by the Western blot procedure (described below). The sample nitrosylation (biotin-conjugated proteins) was visualized by streptavidin-HRP (Abcam, Cambridge, UK, Cat# ab7403, 1:5000).

**Figure 22:**
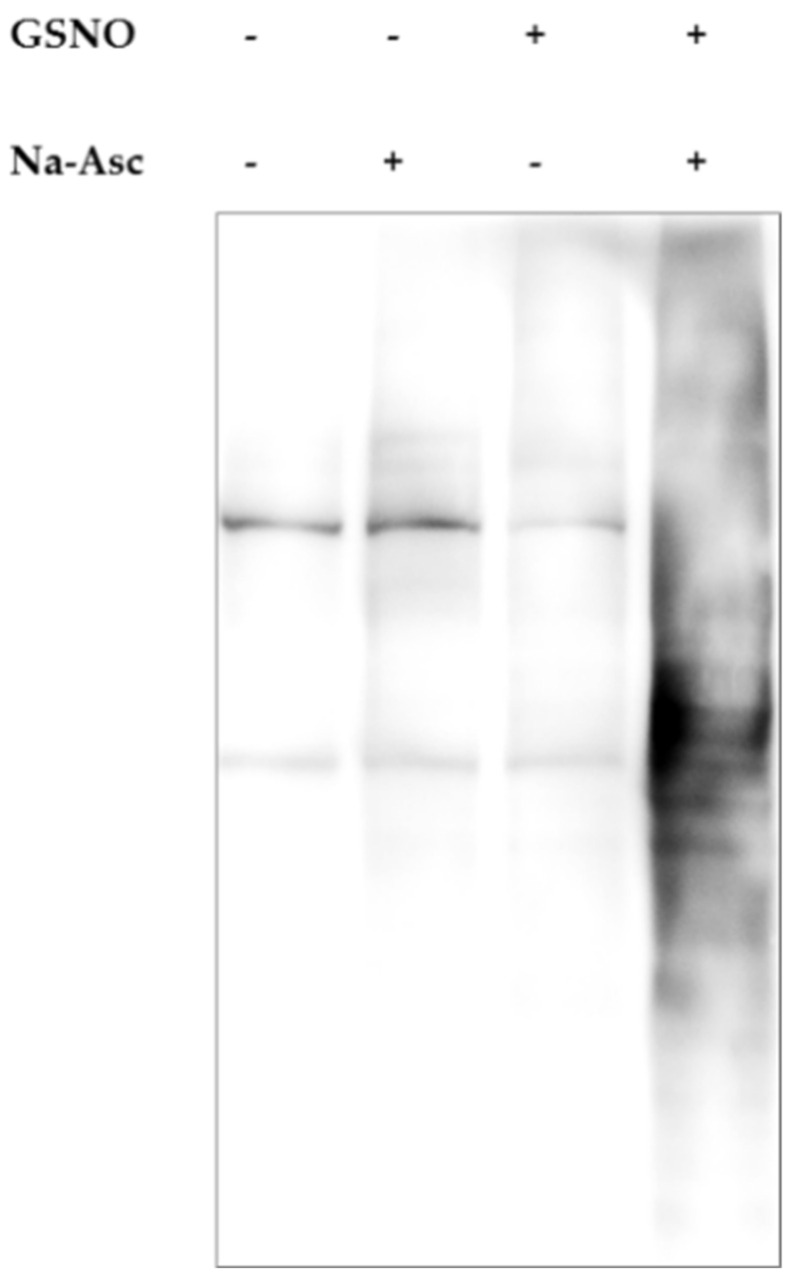
Representative results demonstrating specificity of biotin switch assay. Lysates of the prefrontal cortex were incubated for 30 min with (or in the absence of) 200 μM S-nitrosoglutathione (GSNO), a NO donor. During the labeling step with or without the addition of sodium ascorbate (Na-Asc), biotinylation of the nascent thiols occurred. The sample treated with both GSNO and Na-Asc showed a strong signal compared to other control samples.

Control experimental results showing the specificity of the method are presented in Figure 22.

#### Western Blot Assessment of S-Nitrosylation

A total of 10 µg of total protein obtained from tissue lysates or 20 μL of eluate biotin-conjugated protein was separated on 8% SDS-PAGE gels and transferred to nitrocellulose membranes according to the standard Western blot procedure. After the transfer, the membranes were incubated in TBS-T buffer (Thermo Scientific, Schwerte, Germany) with 5% bovine serum albumin (Fraction V) (Sigma Aldrich, Darmstadt, Germany) at RT for 60 min. Then, incubation with primary antibodies for β-actin (Sigma Aldrich, Darmstadt, Germany, Cat# A5441, 1:10,000), Alzheimer precursor protein A4 (Millipore, Darmstadt, Germany, Cat# MAB348, 1:2000), glutamate transporter 1 (Millipore, Darmstadt, Germany, AB1783, 1:3000) and tau (Abcam, Cambridge, UK, Cat# ab32057, 1:2000) was performed. Incubation was carried out at RT for 2 h. Secondary horseradish peroxidase (HRP)-conjugated antibodies were incubated with membranes at RT for 1 h (Promega, Cat# W402B and Cat# W401B, both 1:5000; Millipore, Cat# AP108P, 1:10,000). Detection was performed using SuperSignal West Pico PLUS blotting substrate (Thermo Scientific, Schwerte, Germany) and the GeneGnome XRQ analysis system (SYNGENE, Cambridge, UK). The data were analyzed using GnomeSys 1.8.2.

### 4.6. cGMP ELISA

Tissues were collected in liquid nitrogen and stored until isolation at −80 °C. The samples were homogenized in 5% TCA in water (half of the prefrontal cortex ~75 mg and hippocampi ~35 mg), followed by centrifugation at 3000× *g* for 5 min. The levels of acetylated cGMP in supernatant from prefrontal cortices and hippocampi were quantified using an ELISA assay (Cayman Chemicals, Tallinn, Estonia, Cat# 581021) according to the manufacturer’s instructions. Under experimental conditions, the standard curve range was 0.023–3 pmol/1000 μL.

### 4.7. Statistics

The statistical significance of the results was determined by one-way ANOVA followed by Tukey’s post hoc comparison using GraphPad Prism (7.0).

## 5. Conclusions

The signaling pathways of NO produce several cellular effects, all of which have been identified to play a role in schizophrenia or AD, including signaling via soluble guanylate cyclase and the cyclic guanosine monophosphate (cGMP) pathway [73] or direct S-nitrosylation of protein cysteine residues (addition of a nitrosyl ion NO^−^ to generate a nitrosothiol, RS-N=O) (reviewed in [61]). Our results show that both MK-801 and scopolamine decrease L-arginine bioavailability, and the impact of MK-801 is more robust, affecting L-arginine levels in both the PFC and hippocampus. Decreased bioavailability of L-arginine may lead to the increased production of ROS/RNS due to NOS uncoupling [74,75,76], and oxidative stress is believed to be involved in the pathology of both schizophrenia and Alzheimer’s disease [77,78].

Both MK-801 and scopolamine exerted a strong detrimental effect on eNOS-derived monomer production, with no significant impact on nNOS, putatively indicating a detrimental influence on BBB permeability. The MK-801-induced effect in this case is more significant. Both compounds induce aberrant S-nitrosylation of GLT-1 transport protein, contributing to disturbed glutamate reuptake.

Diversion of NO signaling depends on the local cellular microenvironment, including levels of transition metal complexes and redox status [79]. It is impossible to establish all of the numerous effects of NO in the multicellular environment of the brain; however, disturbed L-arginine bioavailability due to SDMA/NMMA overproduction, increased eNOS monomer content and aberrant GLT-1 protein nitrosylation seem to underlie the neurochemical basis of the MK-801- or scopolamine-induced effect to some extent, although the impact of compounds varies depending on the structure. The total nNOS expression seems to remain unchanged.

The present results supplement the existing knowledge on the mechanisms of action of MK-801 or scopolamine. To date, the data concerning the impact of the compounds on NO-dependent pathways were incomplete or missing. The search for novel drugs to improve learning and memory is the subject of many studies around the world and the MK-801- or scopolamine-induced memory decline is the most frequently chosen research model. Our results may contribute to better understanding of the mechanisms of novel drugs’ action, in particular their potential to normalize NO-dependent pathways disturbed in pathological processes associated with the cognitive impairments.

## Figures and Tables

**Table 1:** Mass spectrometric parameters for multiple reaction monitoring. Prime transition ions in bold.

Compound	Q1 Mass (Da) Precursor Ion	Q3 Mass (Da) Product Ion	DP (Volts)	CE (Volts)
**SDMA**	**203.30**	**172.10**	**25**	**19**
SDMA	203.30	133.10	25	19
**ADMA**	**203.30**	**45.90**	**15**	**37**
ADMA	203.30	42.90	15	67
**NMMA**	**189.08**	**69.90**	**10**	**33**
NMMA	189.08	116.00	10	21
**L-Arginine**	**175.07**	**70.10**	**30**	**30**
L-Arginine	175.07	116.00	30	20
**L-Citrulline**	**176.06**	**159.10**	**15**	**13**
L-Citrulline	176.06	69.90	15	32
**L-Glutamate**	**148.12**	**84.0**	**10**	**21**
L-Glutamate	148.12	130.0	10	13
**L-Glutamine**	**147.12**	**84.0**	**10.0**	**23**
L-Glutamine	147.12	130.0	10.0	13
**L-Ornithine**	**133.01**	**70.0**	**10.0**	**23**
L-Ornithine	133.01	116.0	10.0	13.0

## Data Availability

Not applicable.

## References

[1] Camina E., Güell F. (2017). The Neuroanatomical, Neurophysiological and Psychological Basis of Memory: Current Models and Their Origins. Front. Pharmacol..

[2] Nursey J., Phelps A.J., Fink G. (2016). Stress, Trauma, and Memory in PTSD. Stress: Concepts, Cognition, Emotion, and Behavior.

[3] Eichenbaum H. (2001). The hippocampus and declarative memory: Cognitive mechanisms and neural codes. Behav. Brain Res..

[4] Warden M.R., Miller E.K. (2010). Task-Dependent Changes in Short-Term Memory in the Prefrontal Cortex. J. Neurosci. Off. J. Soc. Neurosci..

[5] Funahashi S. (2017). Working Memory in the Prefrontal Cortex. Brain Sci..

[6] Miller E.K., Cohen J.D. (2001). An Integrative Theory of Prefrontal Cortex Function. Annu. Rev. Neurosci..

[7] Abraham W.C., Jones O.D., Glanzman D.L. (2019). Is plasticity of synapses the mechanism of long-term memory storage?. NPJ Sci. Learn..

[8] Squire L.R., Genzel L., Wixted J.T., Morris R.G. (2015). Memory Consolidation. Cold Springs Harb. Perspect. Biol..

[9] Javitt D.C. (2010). Glutamatergic theories of schizophrenia. Isr. J. Psychiatry Relat. Sci..

[10] Neill J.C., Barnes S., Cook S., Grayson B., Idris N.F., McLean S.L., Snigdha S., Rajagopal L., Harte M.K. (2010). Animal models of cognitive dysfunction and negative symptoms of schizophrenia: Focus on NMDA receptor antagonism. Pharmacol. Ther..

[11] Bajo R., Pusil S., López M.E., Canuet L., Pereda E., Osipova D., Maestú F., Pekkonen E. (2015). Scopolamine effects on functional brain connectivity: A pharmacological model of Alzheimer’s disease. Sci. Rep..

[12] Kwon S.H., Lee H.K., Kim J.A., Hong S.I., Kim H.C., Jo T.H., Park Y.I., Lee C.K., Kim Y.B., Lee S.Y. (2010). Neuroprotective effects of chlorogenic acid on scopolamine-induced amnesia via anti-acetylcholinesterase and anti-oxidative activities in mice. Eur. J. Pharmacol..

[13] Tang K.S. (2019). The cellular and molecular processes associated with scopolamine-induced memory deficit: A model of Alzheimer’s biomarkers. Life Sci..

[14] Arancio O., Kiebler M., Lee C.J., Lev-Ram V., Tsien R.Y., Kandel E.R., Hawkins R.D. (1996). Nitric Oxide Acts Directly in the Presynaptic Neuron to Produce Long-Term Potentiationin Cultured Hippocampal Neurons. Cell.

[15] Pigott B.M., Garthwaite J. (2016). Nitric oxide is required for L-type Ca^2+^ channel-dependent long-term potentiation in the hippocampus. Front. Synaptic Neurosci..

[16] Choi Y.-B., Tenneti L., Le D.A., Ortiz J., Bai G., Chen H.-S.V., Lipton S.A. (2000). Molecular basis of NMDA receptor-coupled ion channel modulation by S-nitrosylation. Nat. Neurosci..

[17] Ozdemir H., Ertugrul A., Basar K., Saka E. (2012). Differential effects of antipsychotics on hippocampal presynaptic protein expressions and recognition memory in a schizophrenia model in mice. Prog. Neuro-Psychopharmacol. Biol. Psychiatry.

[18] Cieślik P., Woźniak M., Tokarski K., Kusek M., Pilc A., Płoska A., Radulska A., Pelikant-Małecka I., Żołnowska B., Sławiński J. (2019). Simultaneous activation of muscarinic and GABA B receptors as a bidirectional target for novel antipsychotics. Behav. Brain Res..

[19] Cieślik P., Radulska A., Burnat G., Kalinowski L., Wierońska J.M. (2021). Serotonergic–muscarinic interaction within the prefrontal cortex as a novel target to reverse schizophrenia-related cognitive symptoms. Int. J. Mol. Sci..

[20] Cieślik P., Domin H., Chocyk A., Gruca P., Litwa E., Płoska A., Radulska A., Pelikant-Małecka I., Brański P., Kalinowski L. (2020). Simultaneous activation of mGlu2 and muscarinic receptors reverses MK-801-induced cognitive decline in rodents. Neuropharmacology.

[21] Cieślik P., Woźniak M., Rook J.M., Tantawy M.N., Conn P.J., Acher F., Tokarski K., Kusek M., Pilc A., Wierońska J.M. (2018). Mutual activation of glutamatergic mGlu4 and muscarinic M4 receptors reverses schizophrenia-related changes in rodents. Psychopharmacology.

[22] Sałat K., Podkowa A., Mogilski S., Zaręba P., Kulig K., Sałat R., Malikowska N., Filipek B. (2015). The effect of GABA transporter 1 (GAT1) inhibitor, tiagabine, on scopolamine-induced memory impairments in mice. Pharmacol. Rep..

[23] Tain Y.-L., Hsu C.-N. (2017). Toxic Dimethylarginines: Asymmetric Dimethylarginine (ADMA) and Symmetric Dimethylarginine (SDMA). Toxins.

[24] Saigusa D., Takahashi M., Kanemitsu Y., Ishida A., Abe T., Yamakuni T., Suzuki N., Tomioka Y. (2011). Determination of Asymmetric Dimethylarginine and Symmetric Dimethylarginine in Biological Samples of Mice Using LC/MS/MS. Am. J. Anal. Chem..

[25] Hu X., Atzler D., Xu X., Zhang P., Guo H., Lu Z., Fassett J., Schwedhelm E., Böger R.H., Bache R.J. (2011). Dimethylarginine Dimethylaminohydrolase-1 Is the Critical Enzyme for Degrading the Cardiovascular Risk Factor Asymmetrical Dimethylarginine. Arterioscler. Thromb. Vasc. Biol..

[26] Bode-Böger S.M., Scalera F., Kielstein J.T., Martens-Lobenhoffer J., Breithardt G., Fobker M., Reinecke H. (2006). Symmetrical dimethylarginine: A new combined parameter for renal function and extent of coronary artery disease. J. Am. Soc. Nephrol..

[27] Rees D.D., Palmer R.M.J., Hodson H.F., Moncada S. (1989). A specific inhibitor of nitric oxide formation from l-arginine attenuates endothelium-dependent relaxation. Br. J. Pharmacol..

[28] Danysz W., Zajaczkowski W., Parsons C.G. (1995). Modulation of learning processes by ionotropic glutamate receptor ligands. Behav. Pharmacol..

[29] Piedrafita B., Cauli O., Montoliu C., Felipo V. (2007). The function of the glutamate-nitric oxide-cGMP pathway in brain in vivo and learning ability decrease in parallel in mature compared with young rats. Learn. Mem..

[30] Neitz A., Mergia E., Imbrosci B., Petrasch-Parwez E., Eysel U.T., Koesling D., Mittmann T. (2014). Postsynaptic NO/cGMP increases NMDA receptor currents via hyperpolarization-activated cyclic nucleotide-gated channels in the hippocampus. Cereb. Cortex.

[31] Baek K.J., Thiel B.A., Lucas S., Stuehr D.J. (1993). Macrophage nitric oxide synthase subunits. Purification, characterization, and role of prosthetic groups and substrate in regulating their association into a dimeric enzyme. J. Biol. Chem..

[32] Chen W., Xiao H., Rizzo A.N., Zhang W., Mai Y., Ye M. (2014). Endothelial Nitric Oxide Synthase Dimerization Is Regulated by Heat Shock Protein 90 Rather than by Phosphorylation. PLoS ONE.

[33] Frankiewicz T., Potier B., Bashir Z.I., Collingridge G.L., Parsons C.G. (1996). Effects of memantine and MK-801 on NMDA-induced currents in cultured neurones and on synaptic transmission and LTP in area CA1 of rat hippocampal slices. Br. J. Pharmacol..

[34] Hirotsu I., Hori N., Katsuda N., Ishihara T. (1989). Effect of anticholinergic drug on long-term potentiation in rat hippocampal slices. Brain Res..

[35] Leung L.S., Shen B., Rajakumar N., Ma J. (2003). Cholinergic Activity Enhances Hippocampal Long-Term Potentiation in CA1 during Walking in Rats. J. Neurosci. Off. J. Soc. Neurosci..

[36] Knox L.T., Jing Y., Fleete M.S., Collie N.D., Zhang H., Liu P. (2011). Scopolamine impairs behavioural function and arginine metabolism in the rat dentate gyrus. Neuropharmacology.

[37] Nasyrova R.F., Ivashchenko D.V., Ivanov M.V., Neznanov N.G. (2015). Role of nitric oxide and related molecules in schizophrenia pathogenesis: Biochemical, genetic and clinical aspects. Front. Physiol..

[38] Lüth H.-J., Münch G., Arendt T. (2002). Aberrant expression of NOS isoforms in Alzheimer’s disease is structurally related to nitrotyrosine formation. Brain Res..

[39] Balez R., Ooi L. (2016). Getting to NO Alzheimer’s Disease: Neuroprotection versus Neurotoxicity Mediated by Nitric Oxide. Oxid. Med. Cell. Longev..

[40] Conn P.J., Lindsley C.W., Jones C.K. (2009). Activation of metabotropic glutamate receptors as a novel approach for the treatment of schizophrenia. Trends Pharmacol. Sci..

[41] Javitt D.C. (2007). Glutamate and Schizophrenia: Phencyclidine, N-Methyl-d-Aspartate Receptors, and Dopamine-Glutamate Interactions. Int. Rev. Neurobiol..

[42] Moghaddam B., Javitt D. (2012). From revolution to evolution: The glutamate hypothesis of schizophrenia and its implication for treatment. Neuropsychopharmacology.

[43] Hasselmo M.E., Wyble B.P. (1997). Free recall and recognition in a network model of the hippocampus: Simulating effects of scopolamine on human memory function. Behav. Brain Res..

[44] More S.V., Kumar H., Cho D.-Y., Yun Y.-S., Choi D.-K. (2016). Toxin-Induced Experimental Models of Learning and Memory Impairment. Int. J. Mol. Sci..

[45] Tamagnini F., Barker G., Warburton E.C., Burattini C., Aicardi G., Bashir Z.I. (2013). Nitric oxide-dependent long-term depression but not endocannabinoid-mediated long-term potentiation is crucial for visual recognition memory. J. Physiol..

[46] Daniel H., Hemart N., Jaillard D., Crepel F. (1993). Long-term depression requires nitric oxide and guanosine 3′:5′ cyclic monophosphate production in rat cerebellar Purkinje cells. Eur. J. Neurosci..

[47] Calabresi P., Gubellini P., Centonze D., Sancesario G., Morello M., Giorgi M., Pisani A., Bernardi G. (1999). A critical role of the nitric oxide/cGMP pathway in corticostriatal long-term depression. J. Neurosci..

[48] Rozov A.V., Valiullina F.F., Bolshakov A.P. (2017). Mechanisms of Long-Term Plasticity of Hippocampal GABAergic Synapses. Biochemistry.

[49] Babiec W.E., Guglietta R., Jami S.A., Morishita W., Malenka R.C., O’Dell T.J. (2014). Ionotropic NMDA receptor signaling is required for the induction of long-term depression in the mouse hippocampal CA1 region. J. Neurosci..

[50] Scullion S.E., Barker G.R.I., Warburton E.C., Randall A.D., Brown J.T. (2019). Muscarinic Receptor-Dependent Long Term Depression in the Perirhinal Cortex and Recognition Memory are Impaired in the rTg4510 Mouse Model of Tauopathy. Neurochem. Res..

[51] Fish J.E., Marsden P.A. (2006). Endothelial nitric oxide synthase: Insight into cell-specific gene regulation in the vascular endothelium. Cell. Mol. Life Sci..

[52] Förstermann U., Münzel T. (2006). Endothelial nitric oxide synthase in vascular disease: From marvel to menace. Circulation.

[53] Jiang Z., Li C., Arrick D.M., Yang S., Baluna A.E., Sun H. (2014). Role of nitric oxide synthases in early blood-brain barrier disruption following transient focal cerebral ischemia. PLoS ONE.

[54] Santhanam A.V.R., D’Uscio L.V., He T., Das P., Younkin S.G., Katusic Z.S. (2015). Uncoupling of endothelial nitric oxide synthase in cerebral vasculature of Tg2576 mice. J. Neurochem..

[55] Austin S.A., Katusic Z.S. (2016). Loss of Endothelial Nitric Oxide Synthase Promotes p25 Generation and Tau Phosphorylation in a Murine Model of Alzheimer’s Disease. Circ. Res..

[56] Bloom G.S. (2014). Amyloid-β and Tau The Trigger and Bullet in Alzheimer Disease Pathogenesis. JAMA Neurol..

[57] Austin S.A., Santhanam A.V., Hinton D.J., Choi D.-S., Katusic Z.S. (2013). Endothelial nitric oxide deficiency promotes Alzheimer’s disease pathology. J. Neurochem..

[58] Geng J., Wang L., Zhang L., Qin C., Song Y., Ma Y., Chen Y., Chen S., Wang Y., Zhang Z. (2008). Blood-Brain Barrier Disruption Induced Cognitive Impairment Is Associated with Increase of Inflammatory Cytokine. Front. Aging Neurosci..

[59] Bowman G.L., Dayon L., Kirkland R., Wojcik J., Peyratout G., Severin I.C., Henry H., Oikonomidi A., Migliavacca E., Bacher M. (2018). Blood-brain barrier breakdown, neuroinflammation, and cognitive decline in older adults. Alzheimers. Dement..

[60] Pollak T.A., Drndarski S., Stone J.M., David A.S., McGuire P., Abbott N.J. (2018). The blood–brain barrier in psychosis. Lancet Psychiatry.

[61] Nakamura T., Tu S., Akhtar M.W., Sunico C.R., Okamoto S., Lipton S.A. (2013). Aberrant Protein S-nitrosylation in neurodegenerative diseases. Neuron.

[62] Raju K., Doulias P.-T., Evans P., Krizman E.N., Jackson J.G., Horyn O., Daikhin Y., Nissim I., Yudkoff M., Nissim I. (2015). Regulation of brain glutamate metabolism by nitric oxide and S-nitrosylation. Sci. Signal..

[63] Peterson A.R., Binder D.K. (2019). Post-translational Regulation of GLT-1 in Neurological Diseases and Its Potential as an Effective Therapeutic Target. Front. Mol. Neurosci..

[64] Sibson N.R., Dhankhar A., Mason G.F., Behar K.L., Rothman D.L., Shulman R.G. (1997). In vivo 13C NMR measurements of cerebral glutamine synthesis as evidence for glutamate-glutamine cycling. Proc. Natl. Acad. Sci. USA.

[65] Gruetter R. (2002). In vivo 13C NMR studies of compartmentalized cerebral carbohydrate metabolism. Neurochem. Int..

[66] Gropman A.L., Summar M., Leonard J.V. (2007). Neurological implications of urea cycle disorder. J. Inherit. Metab. Dis..

[67] Das I., Khan N.S., Puri B.K., Hirsch S.R. (1996). Elevated endogenous nitric oxide synthase inhibitor in schizophrenic plasma may reflect abnormalities in brain nitric oxide production. Neurosci. Lett..

[68] Gubandru M., Margina D., Tsitsimpikou C., Goutzourelas N., Tsarouhas K., Ilie M., Tsatsakis A.M., Kouretas D. (2013). Alzheimer’s disease treated patients showed different patterns for oxidative stress and inflammation markers. Food Chem. Toxicol..

[69] Selly M.L. (2003). Increased concentrations of homocysteine and asymmetric dimethylarginine and decreased concentrations of nitric oxide in the plasma of patients with Alzheimer’s disease. Neurobiol. Aging.

[70] Mulder C., Wahlund L.-O., Blomberg M., de Jong S., van Kamp G.J., Scheltens P., Teerlink T. (2002). Alzheimer’s disease is not associated with altered concentrations of the nitric oxide synthase inhibitor asymmetric dimethylarginine in cerebrospinal fluid. J. Neural Transm..

[71] Chang F., Flavahan S., Flavahan N.A. (2019). Potential pitfalls in analyzing structural uncoupling of enos: Aging is not associated with increased enzyme monomerization. Am. J. Physiol.-Heart Circ. Physiol..

[72] Forrester M.T., Foster M.W., Benhar M., Stamler J.S. (2009). Detection of protein S-nitrosylation with the biotin-switch technique. Free Radic. Biol. Med..

[73] Denninger J.W., Marletta M.A. (1999). Guanylate cyclase and the ⋅NO/cGMP signaling pathway. Biochim. Biophys. Acta-Bioenerg..

[74] Di Meo S., Reed T.T., Venditti P., Victor V.M. (2016). Role of ROS and RNS Sources in Physiological and Pathological Conditions. Oxid. Med. Cell. Longev..

[75] Sun J., Druhan L.J., Zweier J.L. (2010). Reactive oxygen and nitrogen species regulate inducible nitric oxide synthase function shifting the balance of nitric oxide and superoxide production. Arch. Biochem. Biophys..

[76] Pou S., Pou W.S., Bredt D.S., Snyder S.H., Rosen G.M. (1992). Generation of superoxide by purified brain nitric oxide synthase. J. Biol. Chem..

[77] Malinski T. (2007). Nitric oxide and nitroxidative stress in Alzheimer’s disease. J. Alzheimer’s Dis..

[78] Boll K.M., Noto C., Bonifácio K.L., Bortolasci C.C., Gadelha A., Bressan R.A., Barbosa D.S., Maes M., Moreira E.G. (2017). Oxidative and nitrosative stress biomarkers in chronic schizophrenia. Psychiatry Res..

[79] Thomas D.D., Miranda K.M., Colton C.A., Citrin D., Espey M.G., Wink D.A. (2003). Heme proteins and nitric oxide (NO): The neglected, eloquent chemistry in NO redox signaling and regulation. Antioxid. Redox Signal..

